# 

**Published:** 1877-04

**Authors:** 


*CONTENTS.*

| | Page | | Page |
|---|---|---|---|
| Marvels of Man . . . . | 93 | Farmers and Citizens . . | 105 |
| Recreation . . . . . . . | 95 | Over Eating . . . . . . | 107 |
| Habit . . . . . . . . . . . | 96 | "Going It." . . . . . . | 109 |
| Common Sense . . . . . | 97 | A Dangerous Curiosity . . | 110 |
| Ventilating Chambers . . | 99 | The Feet in Winter . . . | 111 |
| Small-Pox . . . . . . . . | 100 | Baths and Bathing . . . | 112 |
| Dr. Hall's Medicines . . . | 102 | Bare Neck and Arms . . | 113 |
| The Teeth . . . . . . . . | 102 | Sleeping Position . . . . | 114 |
| Sleeping Rooms . . . . . | 104 | Pestilence and Plague . . | 115 |

